# Efficient and Precise CRISPR/Cas9-Mediated MECP2 Modifications in Human-Induced Pluripotent Stem Cells

**DOI:** 10.3389/fgene.2019.00625

**Published:** 2019-07-02

**Authors:** Thi Thanh Huong Le, Ngoc Tung Tran, Thi Mai Lan Dao, Dinh Dung Nguyen, Huy Duong Do, Thi Lien Ha, Ralf Kühn, Thanh Liem Nguyen, Klaus Rajewsky, Van Trung Chu

**Affiliations:** ^1^Department of Gene Technology, Vinmec Research Institute of Stem Cell and Gene Technology, Hanoi, Vietnam; ^2^Immune Regulation and Cancer, Max-Delbrück-Center for Molecular Medicine, Berlin, Germany; ^3^iPS Cell Based Disease Modeling, Berlin Institute of Health, Berlin, Germany

**Keywords:** MECP2 mutations, CRISPR/Cas9, RETT syndrome, homologous recombination, iPSCs

## Abstract

Patients with Rett syndrome (RTT) have severe mental and physical disabilities. The majority of RTT patients carry a heterozygous mutation in methyl-CpG binding protein 2 (MECP2), an X-linked gene encoding an epigenetic factor crucial for normal nerve cell function. No curative therapy for RTT syndrome exists, and cellular mechanisms are incompletely understood. Here, we developed a CRISPR/Cas9-mediated system that targets and corrects the disease relevant regions of the MECP2 exon 4 coding sequence. We achieved homologous recombination (HR) efficiencies of 20% to 30% in human cell lines and iPSCs. Furthermore, we successfully introduced a MECP2^R270X^ mutation into the MECP2 gene in human induced pluripotent stem cells (iPSCs). Consequently, using CRISPR/Cas9, we were able to repair such mutations with high efficiency in human mutant iPSCs. In summary, we provide a new strategy for MECP2 gene targeting that can be potentially translated into gene therapy or for iPSCs-based disease modeling of RTT syndrome.

## Introduction

Rett (RTT) syndrome is a genetic neurodevelopmental disorder found predominantly in female infants. Disease symptoms including cognitive disabilities, decreased coordination and mobility, repetitive movements, and slowed brain growth typically appear after 6 to 18 months of age (Hagberg et al., 1983; Hagberg, 1985; Trevathan and Naidu, 1988). The RTT syndrome is caused by dominant negative mutations in the X-linked transcription factor methyl CpG-binding protein 2 (MECP2). Most mutations occur within one of two functional domains of MECP2: the highly conserved methyl binding domain (MDB) or the transcription repressing domain (TRD) (Rasmussen et al., 1975; Amir et al., 1999). Gene therapy using an adeno-associated viral vector to deliver a functional sequence of the *MECP2* gene *in vivo* has been intensively investigated in mouse models (Sinnett and Gray, 2017). However, this approach induces toxicity and side effects due to the supraphysiological expression of exogenous *MECP2* (Sinnett and Gray, 2017). The study by Guy et al. using conditional MECP2 alleles has shown that by restoring the physiological expression level of *MECP2*, the symptoms of RTT syndrome could be reversed in affected adult mice (Guy et al., 2007; Clarke and Abdala Sheikh, 2018). This study suggests that CRISPR/Cas9-mediated correction of MECP2 mutant alleles is a potential gene therapy for RTT.

The type II CRISPR/Cas9 system is a RNA-guided nuclease providing adaptive immunity in *Streptococcus pyogenes*. In mammalian cells, Cas9 nuclease can be used for editing of genomic sequence by the induction of targeted DNA double-strand breaks (DSBs). The induced DSBs are mostly repaired by the non-homologous end-joining (NHEJ) pathway creating micro-deletions/or insertions (INDELs) or, to a lesser extent, by the homologous recombination (HR) pathway allowing precise genetic manipulations if a repair template is provided (Cong et al., 2013; Hsu et al., 2013; Mali et al., 2013; Chu et al., 2015). Many studies have shown that CRISPR/Cas9-mediated mutagenesis can lead to efficient HR in human iPSCs (Byrne et al., 2015; Zhang et al., 2017; Li et al., 2018).

To our knowledge, there is no previous attempt to correct endogenous MECP2 mutant alleles in human cells using the CRISPR/Cas9 system. Here, we provide a system to repair the mutation hotspots of the MECP2 exon 4. We achieved HR efficiency up to 20% to 30% in human cell lines and iPSCs. To show a proof of principle of our system, we precisely inserted the MECP2^R270X^ mutation into the MECP2 gene in wild-type iPSCs. Finally, using the CRISPR/Cas9 system, we subsequently repaired this mutation successfully in mutant iPSCs. Overall, we provide an efficient strategy for MECP2 correction that is crucial for disease modeling and can potentially be translated into gene therapy of the RTT syndrome.

## Materials and Methods

### Cell Culture and Reagents

HEK293 cells were maintained in DMEM (Gibco) supplied with 10% FBS (Gibco). K562 cells were cultured in RPMI 1640 supplied with 10% FBS and 2 mM l-glutamine (Gibco). Human iPSCs (BCRT cell line) were cultured in E8 flex medium (Gibco) according to the manufacturer’s manual. Cas9 protein was purchased from NEB (M0386S), synthetic sgRNA was purchased from Synthego, and ssODN was ordered from IDT. To generate RNP complex, the Cas9 protein and synthetic sgRNA were mixed at ratio 1:2 and incubated at 25°C for 10 min.

### CRISPR/Cas9 and Donor Vectors Construction

sgRNAs were designed based on CrisprGold software (Chu et al., 2016). Forward and reverse oligos were mixed and phosphorylated individually. Then, annealed oligo duplexes were cloned into the BbsI sites of the CRISPR/Cas9-T2A reporter plasmid (Addgene, 64216). To generate the pMECP2-T2A-mCherry reporter donor vector, the 5′ and 3′ homology arms (HA) were amplified from genomic DNA using Hercules Phusion polymerase (Agilent). The 5′ HA fragment was cloned into XhoI/EcoRI sites of pTV-T2A-mCherry; the 3′ HA fragment was cloned into AsiSI/KpnI sites of the pTV-T2A-mCherry vector. To generate the pMECP2 donor vector, 5-kb MECP2 fragment was amplified from genomic DNA, and at the cleavage site, the silent mutations were added to generate a new PstI recognition site.

### Transfection, Electroporation, and Flow Cytometry

Human HEK293 cells were plated into six-well plates at 1 day before transfection. On the day of transfection, cells were supplied with fresh complete medium, and the DNA was mixed with FuGENE^®^ HD Reagent (Promega) in Opti-MEM (Invitrogen) according to the manufacturer’s introduction. After 15 min of incubation at RT, the mixture was dropped slowly into the well. Next day, the medium was exchanged. The transfected cells were analyzed in different time points. For flow cytometry analysis, HEK293 cells were trypsinized and resuspended in PBS/1% bovine serum albumin (BSA) fluorescence-activated cell sorting (FACS) buffer and analyzed with a Fortessa machine (Becton Dickinson).

For electroporation, human K562 cells were harvested and counted, 2 × 10^5^ cells were resuspended with sgRNA/Cas9 RNP and 100 pmol ssODN in 20 µl electroporation buffer P3 (Lonza) and transferred to a 16-strip cuvette and electroporated using a 4D Nucleofector X unit (Lonza). Then, cells were transferred into the pre-warmed complete medium.

For human BCRT-iPSCs, we used Lipofectamine^®^ 3000 (Life Technologies) according to the manufacturer’s manual. Briefly, 1 day before transfection, iPSCs were placed as small clumps in 500 μg/ml of vitronectin (Life Technologies) precoated plate filled with E8 flex completed medium supplemented with Rock inhibitor (10 µM) (Bio Cat). The next day, the medium was replaced without Rock inhibitor 6 to 8 hours before transfection. Diluted plasmids and lipofectamine were prepared as described in manual. In the case of multiple plasmids, pX330 and targeting vector were used for transfection; we used a 1:1 molar ratio. The medium was changed on the next day to stop the reaction. Two days post-transfection, transfected iPSCs were collected by incubation with Accutase (Merck) for 5 min and the cell pellet was then resuspended in a medium containing E8 Flex complete medium, Rock inhibitor (10 µM), RevitaCell Supplement (Life Technologies), and Gentamycin (Lonza) (FACS-PREP medium). GFP^+^ iPSCs were sorted by FACSAria (Becton Dickinson) and placed into a Vitronectin-precoated plate filled with FACS-PREP medium. The next day, the medium was replaced by E8 flex completed medium. Cells were maintained in culture for several passages before analysis.

For introducing the MECP2^R270X^ mutation into the MECP2 gene, wild-type iPSCs were transfected with 2 μg of the plasmid expressing Cas9 and sgRNA-5 and 60 pmol of ssODN-R270X donor using Lipofectamine^®^ 3000 (Life Technologies). Two days post-transfection, 10^3^ GFP^+^ iPS cells were sorted and plated on a Vitronectin-coated well of a six-well plate in the FACS-PREP medium. The medium was changed the next day. Seven days after sorting, iPSC clones were picked and plated on a new Vitronectin-coated well of a six-well plate. iPSC clones were expanded for 2 weeks. To this end, iPSC clones were harvested for analyzing insertion efficiency by genotyping PCR, RFLP assay, and Sanger sequencing. To repair the MECP2^R270X^ mutation, homozygous mutant iPSC clone (clone 18) was transfected with the plasmid expressing Cas9 and sgRNA-3 together with the donor template plasmid containing silent mutations and a recognition site of PstI restriction enzyme as described above.

### Genomic DNA Isolation, PCR, T7EI, and RFLP Assay

Reporter^+^ cells were cultured and harvested at different time points. Single-cell clones were sorted in 96-well plates. Genomic DNA was extracted using the QuickExtract DNA extraction kit (Epicentre) following the manufacturer’s instruction. For T7EI assay, PCR was done using Herculase II Fusion DNA Polymerase (Agilent Technology) with PCR gene-specific primers (**Table 1**) using the following conditions: 98°C for 3 min; 39 cycles (95°C for 20 s, 60°C for 20 s, 72°C for 20 s), and 72°C for 3 min. PCR products were run on 2% agarose gels, purified, denatured, annealed, and treated with T7EI (NEB). Cleaved DNA fragments were separated on 2% agarose gels, and the DNA concentration of each band was quantified using the ImageJ software. For genotyping RFLP assay, PCR product was purified and digested with a PstI restriction enzyme for 1 h. The digestion was separated on 2% agarose gels.

**Table 1:** Sequences of sgRNAs, ssODNs, and primers were used in this study.

Name	Forward (5′>>3′)	Reverse (5′>>3′)
sg1	CACCGAGAGTTAGCTGACTTTACA	AAACTGTAAAGTCAGCTAACTCTC
sg2	CACCGTTAGCTGACTTTACACGGAG	AAACCTCCGTGTAAAGTCAGCTAAC
sg3	CACCGCTTTTTGGCCTCGGCGGCAG	AAACCTGCCGCCGAGGCCAAAAAGC
sg4	CACCGGGTGGCAGCCGCTGCCGCCG	AAACCGGCGGCAGCGGCTGCCACCC
sg5	CACCGTCAGGCCATTCCCAAGAAAC	AAACGTTTCTTGGGAATGGCCTGAC
MECP2 T7 set 1	CCAGAGGAGGCTCACTGGAGAGCGAC	GCTTGTCTGGTCAGTAGTATCTGCAGC
MECP2 T7 set 2	ACCACATCCACCCAGGTCATGGTGATC	TGGTGATGGTGGTGGTGCTCCTTCTTG
MECP2 HR	TAGAATAGGTAGGGTGCTCTTCTCCACCGG	CGGGATTCTCCTCCACGTCACCGCATGTT
MECP2 RT-PCR	GGAAATCTGGCCGCTCTGCTGGGA	ATTAGGGTCCAGGGATGTGTCGCCT
Gapdh RT-PCR	ATGCATCCTGCACCACCAACTGCTTAG	GGATGCAGGGATGATGTTCTGGGCAGC
ssODN donor	CATTCCCAAGAAACGGGGCCGAAAGCCGGGGAGTGTGGTGGCAGCAGCTGCAGCAGAGGCCAAAAAGAAAGCCGTGAAGGAGTCTTCTATCCGATCTGTG	
ssODN R270X	GGTGATCAAACGCCCCGGCAGGAAGCGAAAAGCTGAGGCCGACCCTCAGGCCATTCCCAAAAAGCGCGGCTGAAATCTAGAGAGTGTGGTGGCAGCCGCTGCCGCCGAGGCCAAAAAGAAAGCCGTGAAGGAGTCTTCTAT	

DNA sequencing PCR products were directly sequenced by specific primers or cloned into the pSTBlue-1 Blunt vector (Novagen) following the manufacturer’s protocol. Plasmid DNAs were isolated using the NucleoSpin Plasmid (Macherey-Nagel). Plasmids were sequenced using T7 forward primer (5′-TAATACGACTCACTATAGGG-3′) by the Sanger method (LGCgenomics, Berlin, Germany).

### qRT-PCR

Total RNA was extracted from wild-type, mutant, and repaired iPSC clones with RNAeasy Mini Kit (Qiagen) and was reverse-transcribed into cDNA with a SuperScript^™^ III kit (Invitrogen). The expression of MECP2 was measured by real-time PCR using SYBR green PCR Master Mix (Thermo Scientific) and StepOnePlus^™^ (Applied Biosystems). The relative expression level of MECP2 was normalized with GAPDH housekeeping gene.

### Statistical Analysis

Statistical tests were performed using Prism 7.0 (GraphPad) using a paired two-tailed Student’s t-test. ****P <0.0001.

## Results

### CRISPR/Cas9-Mediated Reporter Insertion in the MECP2 Locus

**Figure 1:**
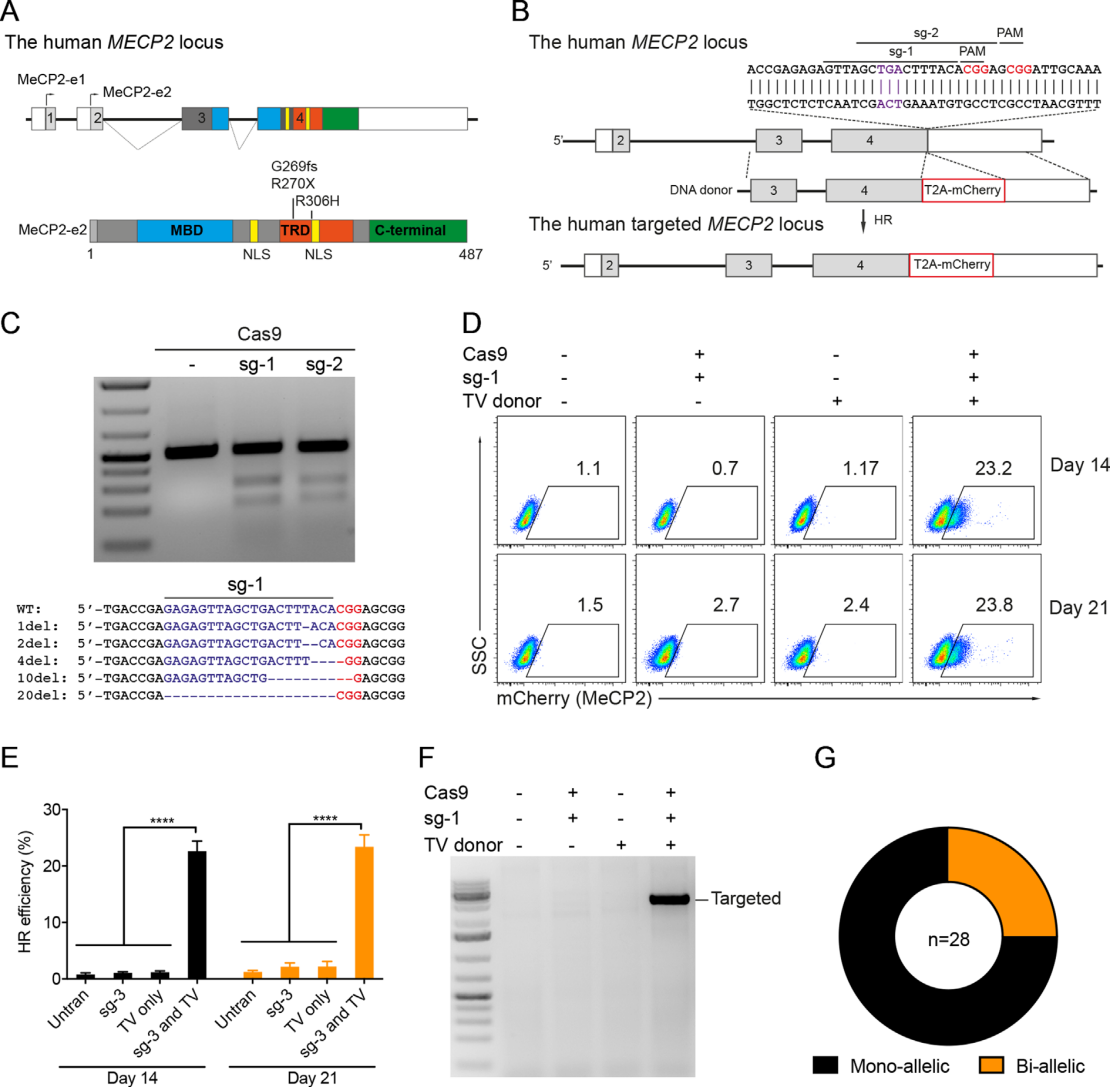
CRISPR/Cas9-mediated reporter insertion in the *MECP2* locus. **(A)** Scheme of human MECP2 isoforms (above) and MECP2 protein with hotspot mutations in exon 4 (below). **(B)** Targeting strategy to insert T2A-mCherry into the MECP2 locus. **(C)** T7EI assay to show the editing activity of sg1 and sg2 (above) in the targeted MECP2 sequence. Sequencing data (below) indicates the INDEL spectrum of sg1 in the targeted MECP2 locus. **(D)** The percentage of mCherry^+^ cells were analyzed by flow cytometry at days 14 and 21 post-transfection. **(E)** The HR efficiency was summarized in the graph from three independent experiments, data show means ± SD (**** p<0.0001). **(F)** Corrected integration PCR. **(G)** Mono-allelic and bi-allelic knockin analysis in single mCherry^+^ cell clones. The data represent at least two independent experiments.

Our previous study identified the mutation spectrum of the MECP2 gene in Vietnamese patients with RTT syndrome. The recurrent mutations T158M, G269fs, R270X, and R306H are located in the MDB and TRD domains of MECP2 protein encoded by exon 4 of the *MECP2* gene (**Figure 1A**). These mutations are specific for the Vietnamese patients, listed in RettBASE (Le Thi Thanh et al., 2018). We first developed a system to quantitatively determine HR efficiency in the human *MECP2* locus by inserting in-frame the coding sequence of cleavage peptide (T2A) and an mCherry reporter in the last exon of the *MECP2* gene. As a result, correctly targeted cells will express the mCherry reporter (**Figure 1B**). We used the CRISPRGold tool (Chu et al., 2016) to design two gRNAs (sg1 and sg2) targeting sequences proximal to the MECP2’s stop codon. T7EI assay indicated that both sgRNAs efficiently targeted the *MECP2* locus upon delivery into HEK293 cells along with Cas9. Sanger sequencing showed a broad range of INDELs at the targeted site (**Figure 1C**). To access HR efficiency, plasmids expressing sg1 and Cas9 were transfected into the HEK293 cells together with donor plasmid carrying the MECP2_T2A_mCherry repair template. Transfected cells were analyzed by flow cytometry at days 14 and 21 post-transfection. We detected about 23% of mCherry^+^ cells in HEK293 cells that received sg1, Cas9, and donor template, but only background signals in control groups transfected with sgRNA/Cas9 or donor template alone (**Figure 1D and E**). We confirmed the corrected integration of the reporter into the targeted *MECP2* locus by using an external forward primer annealing to a genomic sequence outside of the 5′ HA and a specific T2A sequence reverse primer for PCR. The expected ∼3.6-kb fragment was amplified only in HEK293 cells transfected with sg1, Cas9, and donor template (**Figure 1F**). These data indicate the correct configuration of the HR alleles in mcherry^+^ HEK293 cells. Single mCherry^+^ cells were sorted into individual wells of 96-well plates for genotyping PCR. We found that about 75% of these cells harbor heterozygous integrations of the T2A-mCherry sequence into MECP2, whereas homozygous integrations represent about 25% (**Figure 1G**). Taken together, using CRISPR/Cas9 and reporter systems, we achieved efficient HR in the human *MECP2* locus.

### CRISPR/Cas9-Mediated Precise Modification in MECP2 Locus

**Figure 2:**
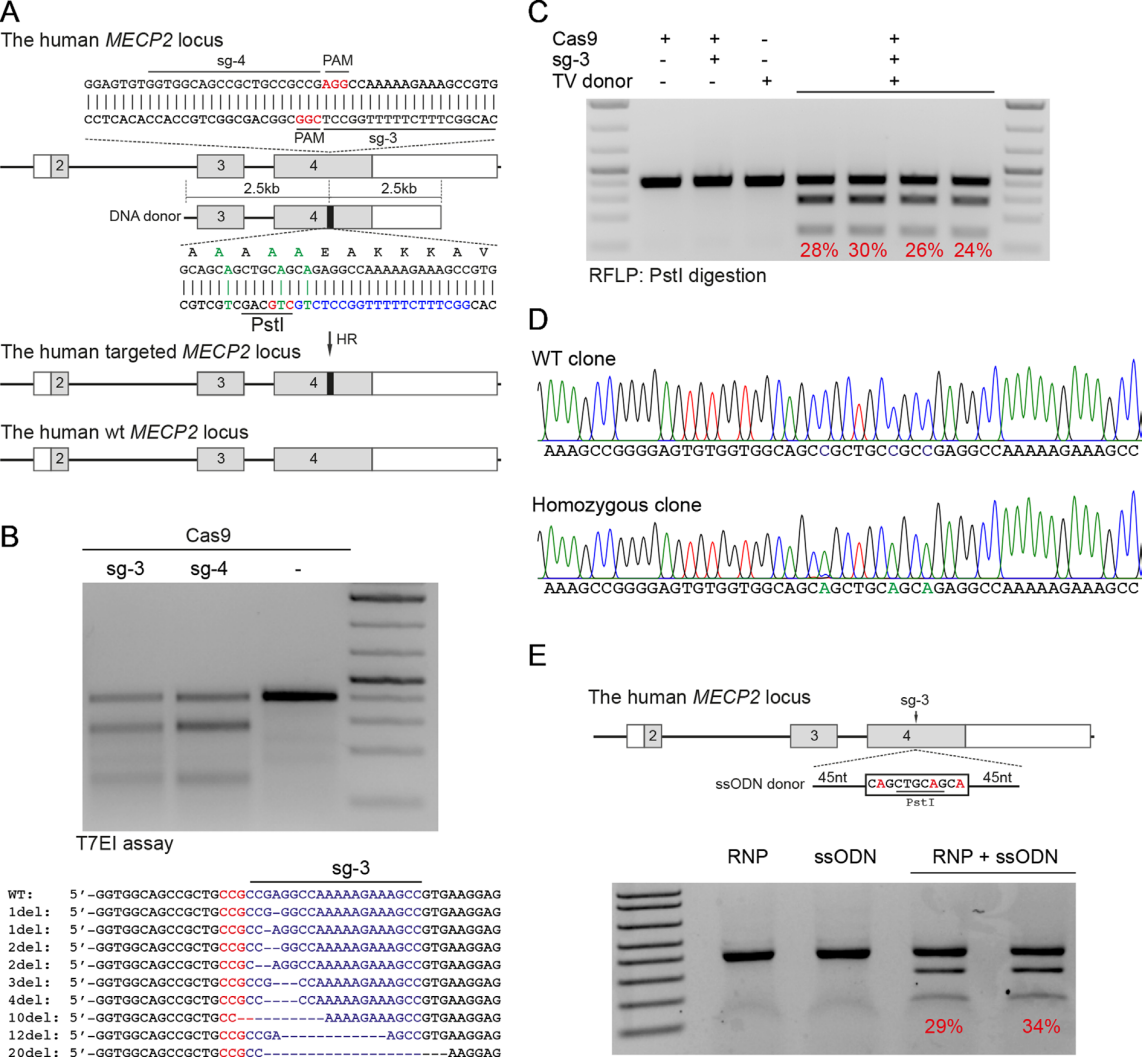
CRISPR/Cas9-mediated efficient precise modification in the *MECP2* locus. **(A)** A strategy to knock-in silent mutations in the MECP2 exon 4. **(B)** T7EI assay showed the cutting efficiencies of two sgRNAs (above) and sequencing data (sg3) showed the INDELs of the targeted MECP2 sequence. **(C)** PstI-mediated restriction fragment length polymorphism (RFLP) assay showed the HR efficiency in HEK cells after 30 days of transfection, numbers indicate HR efficiency. **(D)** Sanger sequencing data of single-cell clone: wild-type (WT) and homozygous targeted clone (below). **(E)** A strategy to knock-in silent mutations in K562 cells using CRISPR/Cas9 RNP and ssODN as a repair template (above) and the RFLP assay to access the knock-in efficiency. Numbers in red represent for the band quantification-based knock-in efficiency. The data represent at least two independent experiments.

Next, we developed the CRISPR/Cas9-mediated system to correct the recurrent mutations in the MECP2 exon 4: G269fs, R270X, and R306H. We designed two sgRNAs close to these mutations (sg3 and sg4) and a repair template containing HAs of 2.5 kb. To facilitate quantification of the HR efficiency, a PstI restriction site was created by introducing silent mutations into the repair template (**Figure 2A**). High editing activities of sg3 and sg4 were validated in HEK293 cells by T7E1 assays (**Figure 2B**, top panel). Sequencing data of sg3-targeted cells showed a broad range of INDELs (**Figure 2B**, bottom panel). Next, plasmids, carrying Cas9/sg3 and donor template were transfected into HEK293T cells. Thirty days post-transfection, genomic DNA of transfected cells was isolated, and the targeted region was amplified by PCR. PstI-mediated restriction fragment length polymorphism (RFLP) showed that PstI-cleaved bands were only detected in cells co-transfected with sg3, Cas9, and repair template. Band quantification showed HR efficiency of 20% to 30% in the *MECP2* locus (**Figure 2C**). Sequencing data of the targeted homozygous clone confirmed that the precise modifications were correctly inserted into the MECP2 gene (**Figure 2D**).

It is known that HR efficiency is determined by many factors. The type of donor template is considered as the most important one (Song and Stieger, 2017). Double-stranded DNA plasmid, PCR sequences, and ssDNA oligonucleotides (ssODN) are often used as a donor template for precise insertion of large or small sequence changes at CRISPR/Cas9-induced DSBs. To test whether we can also use ssODN as donor template for MECP2 precise modifications, we designed a 100-nucleotide ssODN with homology regions of 45 nucleotides each and silent replacements to create a PstI restriction site (**Figure 2E**). Next, we electroporated the sg3/Cas9 RNP complexes with ssODN into human leukemic K562 cells. Four days post-targeting, the targeted cells were harvested for analyzing HR efficiency. As shown in **Figure 2E**, we detected PstI-cleaved PCR products only in cells that received both RNP and ssODN. The HR efficiency ranged from 29% to 34% (**Figure 2E**).

### CRISPR/Cas9-Mediated Reporter Insertion in Human iPSCs

**Figure 3:**
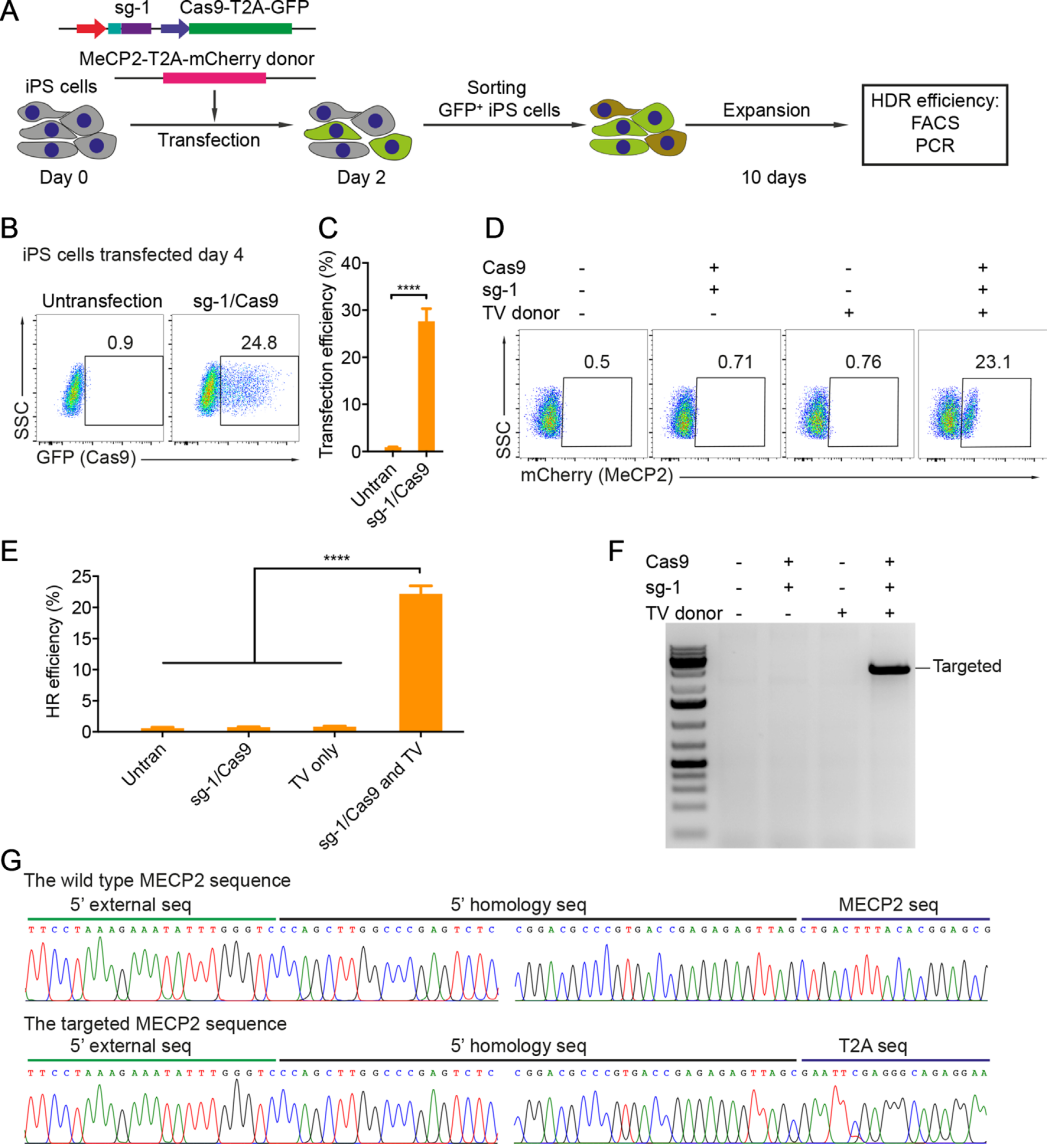
CRISPR/Cas9-mediated reporter insertion in human iPSCs. **(A)** Experimental scheme of gene editing in human iPSCs. **(B)** Transfection efficiency in iPSCs using Lipofectamine 3000 was analyzed by FACS. **(C)** A bar graph indicated the transfection efficiency of human iPSCs, data show means ± SD (****p < 0.0001). **(D)** FACS analysis showed the knock-in efficiency in human iPSCs represented by the mCherry^+^ cells. **(E)** A bar graph showed knock-in efficiencies in human iPSCs, data show means ± SD (****p < 0.0001). **(F)** Corrected integration PCR and Sanger sequencing data showed the junctions of 5′ HA. The data represent at least two independent experiments.

Patient-derived iPSCs have been intensively studied for disease modeling, drug discovery, and potential somatic cell therapy. Thus, we next tested whether our system works efficiently in human iPSCs. We exploited the reporter system as described above to evaluate HR efficiency. The sg1/Cas9 vector and donor template vector were co-transfected into human iPSCs. Two days later, the transfected iPSCs were enriched and expanded (**Figure 3A**). The transfection efficiency in human iPSCs was about 25% to 28% (**Figure 3B and C**). Ten days after expansion, the percentage of mCherry^+^ iPSCs was analyzed by flow cytometry. As shown in **Figure 3D and E**, the percentage of mCherry^+^ iPSCs was about 20% when transfected with sg1/Cas9 and donor template vectors, whereas there were only background signals in control cells transfected with only sg1/Cas9 or donor template vectors alone (**Figure 3D and E**). Correct integration PCR proved that the T2A-mCherry reporter was successfully inserted into the *MECP2* locus in human iPSCs (**Figure 3F**). Sequencing data confirmed that the targeted MECP2 sequences were configured as planned (**Figure 3G**). Overall, we provide a new efficient system to precisely modify the human *MECP2* gene in human iPSCs.

### CRISPR/Cas9-Based Modeling MECP2^R270X^ Mutation in Human iPSCs

**Figure 4:**
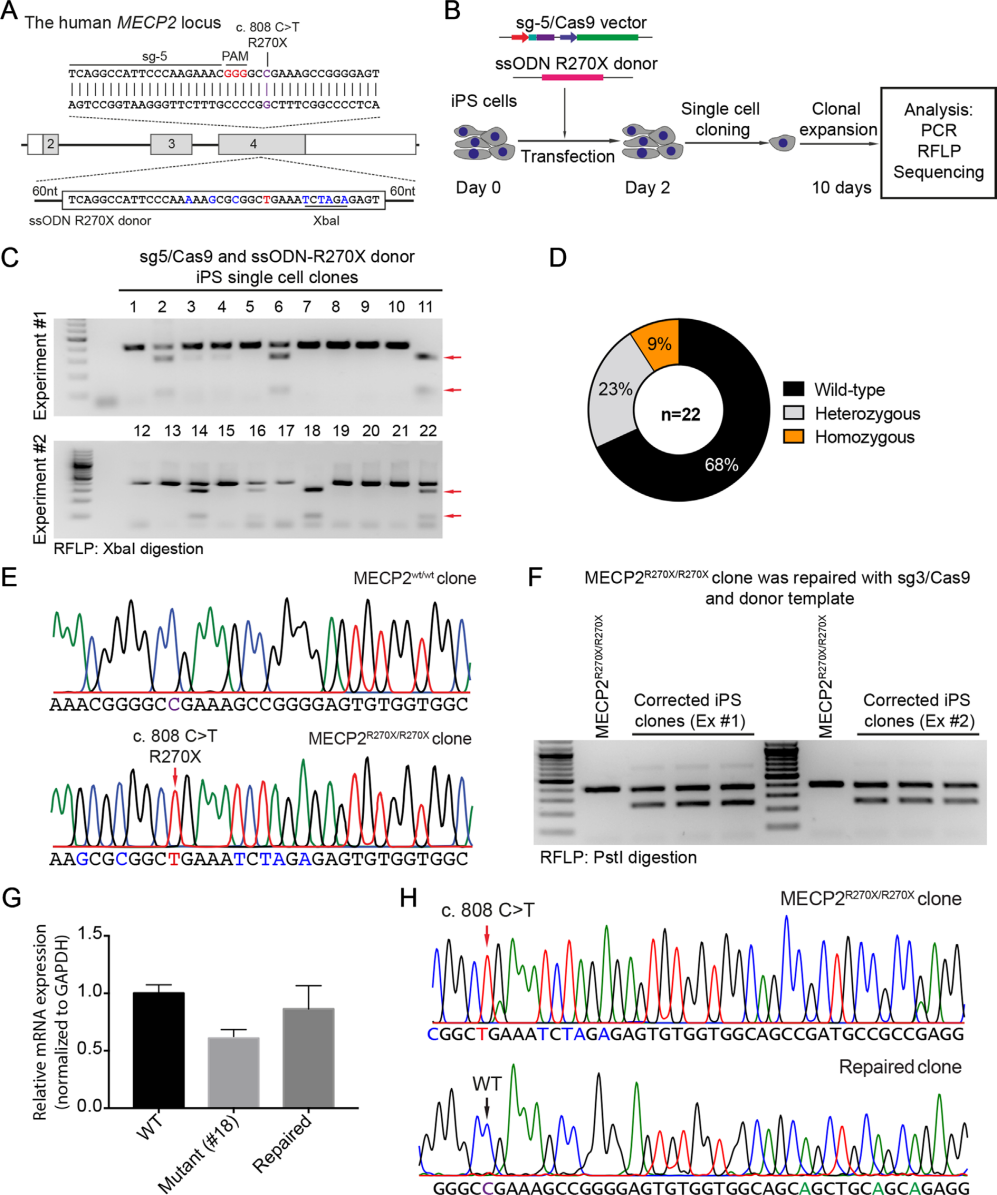
CRISPR/Cas9-mediated insertion of the MECP2^R270X^ mutation in human iPSCs. **(A)** Strategy to insert a c.808 C>T mutation into the *MECP2* gene using CRISPR/Cas9 system. **(B)** Experimental scheme of the MECP2^R270X^ mutation insertion in human iPSCs. **(C)** XbaI-mediated RFLP assay shows heterozygous and homozygous MECP2^R270X^ mutant iPSC clones. **(D)** The donut chart summarizes the data shown in **(C)**. **(E)** Sanger sequencing data confirms insertion of the MECP2^R270X^ mutation in homozygous mutant iPSC clone. **(F)** PstI-mediated RFLP assay shows successful correction of the MECP2^R270X^ mutation in the repaired IPSC clones. **(G)** qRT-PCR data shows the expression level of MECP2 in the wild-type, homozygous mutant, and CRISPR/Cas9-repaired iPSC clones. **(H)** Sanger sequencing data of MECP2 cDNA confirms the MECP2^R270X^ correction in the repaired iPSCs. The data represent at least two independent experiments.

To precisely introduce a MECP2^R270X^ mutation (c.808 C>T) into the *MECP2* gene in human iPSCs, we designed a new sgRNA (sg5) and ssODN (ssODN-R270X) with homology regions of 60 nucleotides, including the C>T mutation and an XbaI recognition site (**Figure 4A**). Next, we transfected human iPSCs with the sg5/Cas9 vectors and ssODN-R270X donor template. Two days post-transfection, the transfected iPCSs were sorted and single cell-derived clones were expanded (**Figure 4B**). To this end, cell clones were harvested for analyzing the insertion efficiency of the MECP2^R270X^ mutation. Among 22 cell clones, we detected two (9%) homozygous and five (23%) heterozygous clones (**Figure 4C and 4D**). Sequencing data confirmed that we succeeded to insert the R270X mutation into the *MECP2* gene in human iPSCs (**Figure 4E**). To show a proof of principle of our correction system, we used the strategy depicted in **Figure 2A**. The sg3/Cas9 and donor template vectors were transfected into MECP2^R270X/R270X^ iPS cells (clone 18, see **Figure 4C**). Two days later, single cell-derived clones were isolated and expanded for 2 weeks. As shown in **Figure 4F**, the PstI-mediated RFLP assay showed that we were able to correct the MECP2^R270X^ mutation in mutant iPSCs. To assess the expression of the MECP2 gene, we isolated mRNA from the wild-type, mutant, and repaired iPSC clones. Quantitative RT-PCR data showed that we enabled to restore the expression level of the MECP2 gene in the repaired iPSCs as comparable as the wild-type iPSCs (**Figure 4G**). Sequencing data of MECP2 cDNA confirmed that the mutant MECP2 allele was successfully repaired in the corrected iPSCs (**Figure 4H**). Thus, this system will open new avenues to iPSC-based disease modeling and therapeutic development of RTT syndrome.

## Discussion

In this study, we developed an efficient CRISPR/Cas9-mediated system that precisely modifies the human *MECP2* locus. Restoration of the physiological expression level of the MECP2 gene is critical for the reversion of disease symptoms. Thus, the correction of mutations in the endogenous MECP2 gene holds promise for gene therapy of RTT syndrome. Using preclinical mouse models of RTT, many laboratories have attempted to deliver intact MECP2 cDNA *in vivo*, using the AAV vectors (Sinnett and Gray, 2017). Although AAV-mediated MECP2 delivery leads to positive effects, such as increasing survival and improving weight, this system still causes multiple side effects and toxicity to animals due to the uncontrolled expression level of the MECP2 transgene (Collins et al., 2004; Luikenhuis et al., 2004; Gadalla et al., 2013). Thus, the expression level of MECP2 is essential for restoring the function of defected neurons. Too much MECP2 protein is as harmful to neural cells as too little. It has been shown that patients with the MECP2 duplication syndrome have two copies of the *MECP2* gene on their single X-chromosome leading to mental disability and autistic-like behavior (Moretti and Zoghbi, 2006). Furthermore, a reduction of MECP2 protein due to protein instability is also related to the milder neurological and psychiatric symptoms, such as anxiety and depression (Ramocki et al., 2009; Chao and Zoghbi, 2012). CRISPR/Cas9-mediated mutation correction restores the endogenous expression of the MECP2 gene that might fully recover RTT symptoms. This system can be potentially applied as gene therapy for RTT patients *in vivo*. However, many challenges remain. For example, the mainly affected cells are neurons that are non-dividing. It is known that the HR pathway occurs in the S-G2 phase of the cell cycle. Thus, non-dividing or low proliferating cells, such as neurons, are not suitable for HR-mediated mutant correction. However, this issue can be potentially addressed by either suppressing the NHEJ pathway or activating HR-related factors (Chu et al., 2015; Charpentier et al., 2018). In addition, the safety of the system needs to be further investigated.

In addition, many studies have shown that MECP2 is not only essential for the functions of neurons but also important for the functions of glial subtypes in the brain as well as of many cell types in the immune system, including microglia and macrophages (Maezawa and Jin, 2010; Derecki et al., 2012; O’Driscoll et al., 2013; Cronk et al., 2015; Jin et al., 2015; Cronk et al., 2016). Physiological restoration of normal MEPC2 functions in neurons and in all immune cells should be considered as an optimal therapy. It is supported that hematopoietic stem cell transplantation (HSCT) is beneficial in Mecp2-deficient mice and that MeCP2 plays important roles for immune cells both in the brain and in the periphery (Maezawa and Jin, 2010; Derecki et al., 2012; O’Driscoll et al., 2013; Cronk et al., 2015; Jin et al., 2015). Thus, correction of the MECP2 mutations *ex vivo* with the CRISPR/Cas9 system followed by autologous HSCT is a promising future therapeutic option for RTT syndrome.

Our system also works efficiently in human iPSCs opening great avenues for disease modeling, drug screening, and somatic cell therapy. The generation of RTT patient-derived iPSCs (RTT-hiPSCs) has been reported by many groups; however, the natural random X-chromosome inactivation (XCI) status of RTT-hiPSCs is inconsistent. XCI results in cellular mosaicism where some cells express wild-type MECP2 (isogenic), whereas other cells express mutant MECP2. Some studies showed that the maintenance of inactive X-chromosome of the founder cell allows RTT-hiPSCs to express either wild-type or mutant MECP2 allele (Ananiev et al., 2011; Cheung et al., 2011; Pomp et al., 2011). In contrast, others reported that the inactive X-chromosome of the founder cells is reactivated during the reprogramming process (Kim et al., 2009; Marchetto et al., 2010). Importantly, RTT-hiPSCs could differentiate into neurons that exhibit disease phenotypes of RTT syndrome. Thus, these cells will be valuable for being rescued by CRISPR/Cas9-mediated MECP2 correction. Using CRISPR/Cas9 technology, we have achieved efficient precise MECP2 modifications in human iPSCs. Overall, our work provides an efficient system for repairing MECP2 mutations in RTT-hiPSCs.

## Data Availability Statement

All datasets generated/analyzed in this study are included in the manuscript or supplementary files.

## Author Contributions

TTHL and VTC designed the project. TTHL, NTT, TMLD, DDN, HDD, TLH, and VTC performed experiments and acquired the data. NTT, VTC, RK, KR, and TLN analyzed and interpreted the data. VTC, NTT, RK, and KR wrote the article.

## Funding

This work was supported by Vinmec Healthcare System (ISC.17.05 to TTHL and TLN).

## Conflict of Interest Statement

The authors declare that the research was conducted in the absence of any commercial or financial relationships that could be construed as a potential conflict of interest.
